# Association of genetic polymorphisms in genes involved in Ara-C and dNTP metabolism pathway with chemosensitivity and prognosis of adult acute myeloid leukemia (AML)

**DOI:** 10.1186/s12967-018-1463-1

**Published:** 2018-04-10

**Authors:** Ke-Wei Zhu, Peng Chen, Dao-Yu Zhang, Han Yan, Han Liu, Li-Na Cen, Yan-Ling Liu, Shan Cao, Gan Zhou, Hui Zeng, Shu-Ping Chen, Xie-Lan Zhao, Xiao-Ping Chen

**Affiliations:** 1 Department of Clinical Pharmacology, Xiangya Hospital, Central South University, Changsha, 410078 People’s Republic of China; 2 Institute of Clinical Pharmacology, Central South University, Hunan Key Laboratory of Pharmacogenetics, Changsha, 410078 People’s Republic of China; 3 National Clinical Research Center for Geriatric Disorders, Xiangya Hospital, Central South University, Changsha, 410008 Hunan People’s Republic of China; 4 Department of Hematology, Xiangya Hospital, Central South University, Changsha, 410078 People’s Republic of China; 5 Department of Clinical Pharmacology, Xiangya Hospital, Central South University, Changsha, 410008 Hunan China

**Keywords:** Adult acute myeloid leukaemia (AML), Cytarabine arabinoside (Ara-C), Single nucleotide polymorphisms (SNPs)

## Abstract

**Background:**

Cytarabine arabinoside (Ara-C) has been the core of chemotherapy for adult acute myeloid leukemia (AML). Ara-C undergoes a three-step phosphorylation into the active metabolite Ara-C triphosphosphate (ara-CTP). Several enzymes are involved directly or indirectly in either the formation or detoxification of ara-CTP.

**Methods:**

A total of 12 eQTL (expression Quantitative Trait Loci) single nucleotide polymorphisms (SNPs) or tag SNPs in 7 genes including *CMPK1*, *NME1*, *NME2*, *RRM1*, *RRM2*, *SAMHD1* and *E2F1* were genotyped in 361 Chinese non-M3 AML patients by using the Sequenom Massarray system. Association of the SNPs with complete remission (CR) rate after Ara-C based induction therapy, relapse-free survival (RFS) and overall survival (OS) were analyzed.

**Results:**

Three SNPs were observed to be associated increased risk of chemoresistance indicated by CR rate (*NME2* rs3744660, *E2F1* rs3213150, and *RRM2* rs1130609), among which two (rs3744660 and rs1130609) were eQTL. Combined genotypes based on *E2F1* rs3213150 and *RRM2* rs1130609 polymorphisms further increased the risk of non-CR. The *SAMHD1* eQTL polymorphism rs6102991 showed decreased risk of non-CR marginally (*P *= 0.055). Three SNPs (*NME1* rs3760468 and rs2302254, and *NME2* rs3744660) were associated with worse RFS, and the *RRM2* rs1130609 polymorphism was marginally associated with worse RFS (*P *= 0.085) and OS (*P *= 0.080). Three SNPs (*NME1* rs3760468, *NME2* rs3744660, and *RRM1* rs183484) were associated with worse OS in AML patients.

**Conclusion:**

Data from our study demonstrated that SNPs in Ara-C and dNTP metabolic pathway predict chemosensitivity and prognosis of AML patients in China.

**Electronic supplementary material:**

The online version of this article (10.1186/s12967-018-1463-1) contains supplementary material, which is available to authorized users.

## Background

Acute myeloid leukemia (AML) is a hematological malignancy characterized by malignant proliferation of the hemopoietic system. AML is a heterogeneous collection of diseases characterized by distinct morphological, chromosomal and cytogenetic abnormalities. Subtype specific therapy for AML is not available despite the FAB M3 subtype. Cytarabine arabinoside (Ara-C) remains the first-line chemotherapeutic agent for the treatment of AML for decades [1–3]. Clinical studies have demonstrated that the complete remission (CR) rate ranges in 50–70% and 5 year survival rate ranges in 27–40% in AML patients receiving Ara-C based chemotherapy [4–6].

Ara-C is a nucleotide analog that is transmembrane transported into leukemia cells by the nucleoside transporters include the solute carrier family 29 member 1 (*SLC29A1*) [7]. Intracellular Ara-C undergoes a three-step phosphorylation into the activate metabolite Ara-C triphosphate (ara-CTP) by deoxycytidine kinase (*DCK*) [8], cytidine monophosphate kinase 1 (*CMPK1*) [9], and nucleoside diphosphate kinases (NDPKs) in turn [10]. Ara-CTP competes with deoxycytidine triphosphate (dCTP) for incorporation into DNA, and thus results in blockade of DNA synthesis and cell death [11]. More recently, Schneider C and colleagues found that ara-CTP is hydrolyzed by the deoxynucleoside triphosphate (dNTP) triphosphohydrolase SAM domain and HD domain 1 (*SAMHD1*), which promotes the detoxification of intracellular ara-CTP pools [12]. Disruption of *SAMHD1* increases Ara-C sensitivity in both cultured leukemia cells and mouse model of AML [13].

**Fig. 1:**
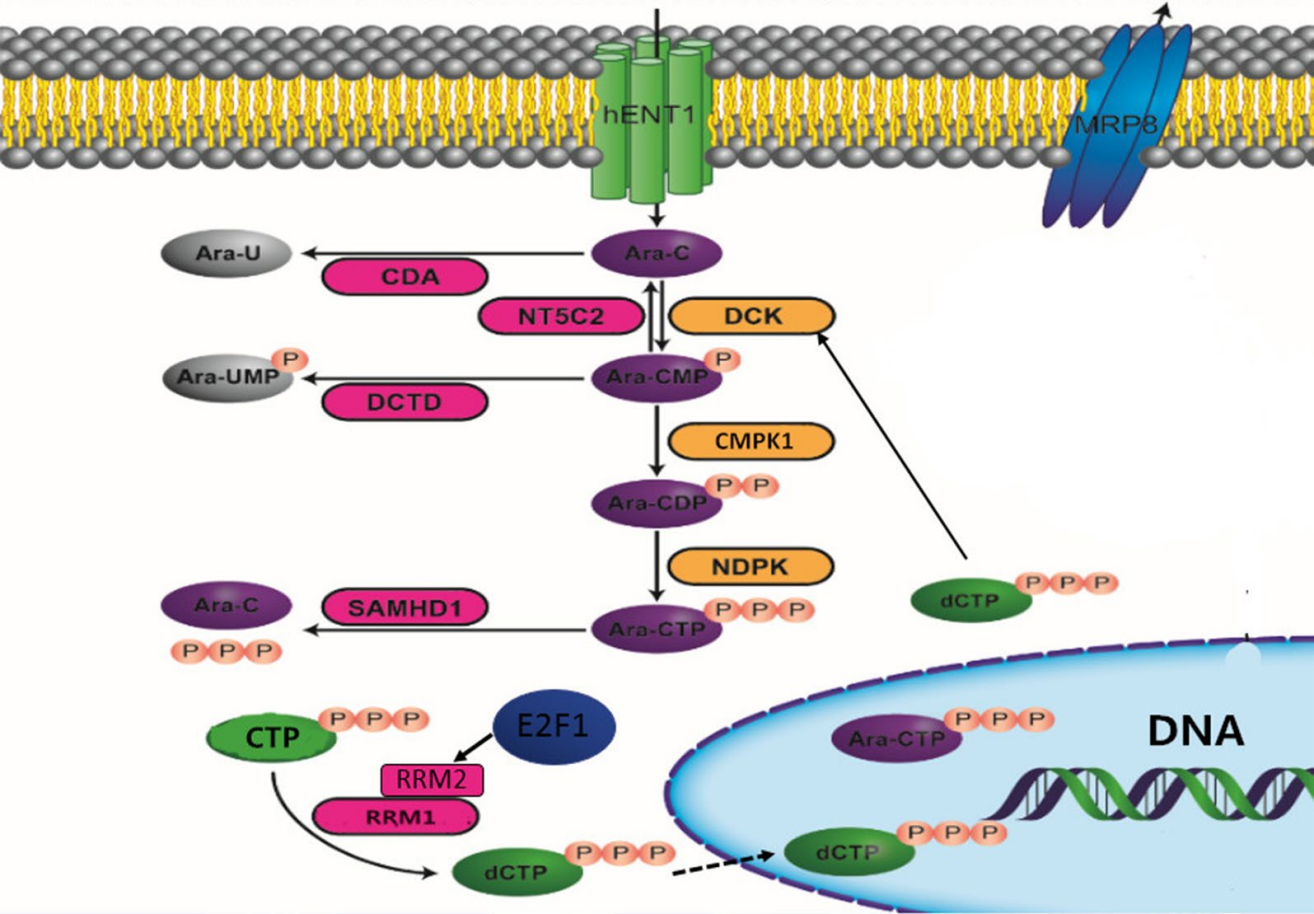
Schematic diagram of the Ara-C metabolizing pathway

Intracellular level of dNTPs, which are consisted of dCTP, dATP, dTTP and dGTP, is another potential mechanism that affect Ara-C sensitivity. High intracellular dNTPs can inhibit Ara-C phosphorylation and decrease the accumulation of ara-CTP through inhibition of *DCK* activity by a feedback mechanism. Exhaustion of the intracellular CTP/dCTP pools facilitates Ara-C phosphorylation and increases the incorporation of ara-CTP into DNA by reducing the feedback inhibition on *DCK* [14]. Ribonucleotide reductase (RR) is a key enzyme responsible for the reduction of ribonucleotides to deoxyribonucleotides and plays important roles in the regulation of intracellular CTP/dCTP pools [15, 16] (Fig. 1). RR is mainly composed of RRM1 and RRM2 subunits, and the expression of *RRM2* is regulated by the transcription factor *E2F1* [17]. An inverse correlation between *RRM1*/*RRM2* mRNA expression and intracellular ara-CTP in leukemia blasts after Ara-C treatment is observed in clinic. Ex vivo ara-C sensitivity study with primary AML samples has also shown correlationship between *RRM1* mRNA expression and Ara-C sensitivity [18]. In addition, higher *RRM1* expression in leukemia blast cells predicts better relapse-free survival (RFS) in AML patients [19].

Influence of genetic factors on either Ara-C chemosensitivity and/or AML prognosis has raised much interest in recent years [20, 21]. Our previous studies have shown associations of single nucleotide polymorphisms (SNPs) in *DCK*, Wilms tumor 1 (*WT1*) and DNA methyltransferase 3 alpha (*DNMT3A*) with drug response to Ara-C based induction therapy in AML [22–24]. In addition, somatic mutations in genes such as *DNMT3A*, FMS-like Tyrosine Kinase3 (*FLT3*) and nucleophosmin (*NPM1*) can also affect AML prognosis [24–26]. For the Ara-C metabolic pathway genes, a promoter polymorphism rs2302254 in *NME1* (encoding NDPK-A) is associated increased risk of Ara-C associated neurotoxicity [27]. The *RRM1* 3′-UTR SNP rs1042919 and promoter SNP rs1561876 are reported to be associated with decreased intracellular ara-CTP levels during Ara-C therapy, and increased risk of relapse and/or worse event free survival (EFS) in Caucasian AML patients treated with cytarabine and cladribine [18]. More recently, association of *RRM1* rs9937 variant with induction therapy related death and survival rates in AML patients is also reported [20]. In the study by Cao et al. the *RRM2* nonsynonymous SNP rs1130609 (S59A) is associated with decreased Ara-C cytotoxicity in HapMap lymphoblast cell lines from the CEU population and poorer EFS for the St Jude AML97 cohort, but the clinical association was not replicated in St Jude AML02 cohort [18]. There is no studies focused on the association of *CMPK1*, *NME2, SAMHD1* and *E2F1* polymorphisms with Ara-C response in AML patients presently.

In this study, we sought to identify genetics polymorphisms in Ara-C and dNTP metabolic pathway including *CMPK1*, *NME1*/*NME2* (encoding NDPK), *SAMHD1*, *RRM1*, *RRM2* and *E2F1* with drug response to Ara-C based chemotherapy and AML prognosis in Chinese AML patients.

## Methods

### Patients

Three hundred and sixty-one newly diagnosed AML patients were recruited from Xiangya hospital from May 2009 to Nov 2017. Peripheral venous blood or bone marrow samples were obtained before chemotherapy. Patients with AML FAB M3 subtype, or combined critical illness and cancer, less than 16 years old, and secondary leukemia were excluded. M3 subtype was excluded because this subtype of patients had unique chemotherapy regimens.

All patients received an “7 + 3” induction regimen consisting of Ara-C (100–200 mg/m^2^ for day 1–7) and anthracyclines (mitoxantrone 8–16 mg/m^2^, or daunorubicin 45–60 mg/m^2^, or aclarubicin 20 mg/m^2^, or pirarubicin 30 mg/m^2^, or idarubicin 10–20 mg/m^2^, for day 1–3). One or two courses of induction chemotherapy were given to obtain complete remission (CR). Achievement of CR was defined according to the international recommendations as we described previously [23], which include: < 5% blasts in the bone marrow; absence of extramedullary disease; absolute neutrophil count > 1.0 × 10^9^/L; platelet count > 100 × 10^9^/L. Once CR was achieved, the patients received sequential consolidation therapy consisting of Ara-C and anthracyclines or haematopoietic stem cell transplantation (HSCT). Regular follow-up was carried out by outpatient review or telephone.

The clinical and pathological information of the AML patients were obtained by chart review of electronic medical record (EMR) from the hospital. Overall survival (OS) and relapse free survival (RFS) were used to indicate disease outcomes. Relapse was defined as the presence of > 5% of blast cells in the bone marrow or reappearance of blast cells in the peripheral blood or development of extramedullary disease. RFS was calculated from the date of achievement of CR until the date of relapse or death from any cause. OS was calculated from the date of AML diagnosis to the date of death from any cause. For those patients without relapse or death event by the end of the study follow-up, survival end-points were censored at the date of last follow-up. The risk stratification criteria based on cytogenetics and molecular characteristics was described elsewhere [23].

### SNPs selection and genotyping

**Fig. 2:**
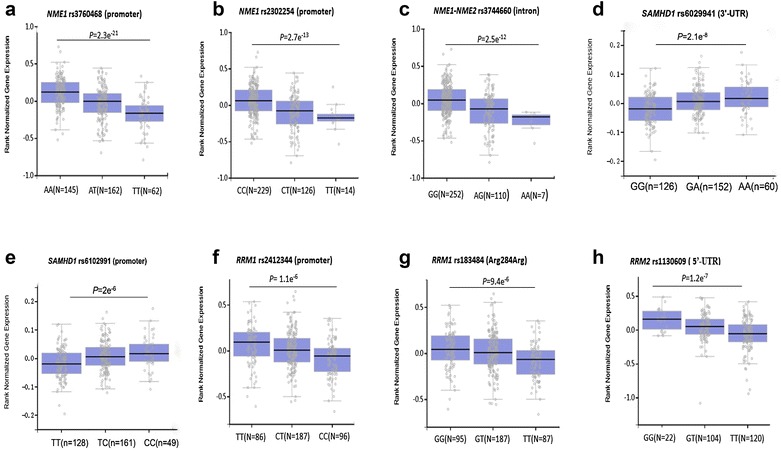
eQTL analysis of 8 candidate SNPs selected in the study. **a** *NME1* rs3760468; **b** *NME1* rs2302254; **c** *NME1*–*NME2* rs3744660; **d** *SAMHD1* rs6029941; **e** *SAMHD1* rs6102991; **f** *RRM1* rs2412344; **g** *RRM1* rs183484; **h** *RRM2* rs1130609

Based on literature searching on Ara-C metabolism related genes in the PubMed database, 7 genes involved in Ara-C metabolic pathway (*CMPK1*, *NEM1*, *NEM2*, *SAMHD1*, *RRM1*, *RRM2*, *E2F1*) were selected. Candidate tag SNPs in these genes were initially selected based on the database from NCBI (https://www.ncbi.nlm.nih.gov/snp/) and the 1000 Genomes Project (https://www.ncbi.nlm.nih.gov/variation/tools/1000 genomes/) using the Haploview 4.2 (Cambridge, MA, USA). Then, potential influence of the SNPs on expression the corresponding genes was analyzed by the expression quantitative trait locus (eQTL) database (https://gtexportal.org/home/). A total of 12 SNPs (*CMPK1* rs7543016, *NME1* rs3760468 and rs2302254, *NME2* rs3744660, *SMAD1* rs6102991, rs28372906 and rs6029941, *E2F1* rs3213150 and rs3213180, *RRM1* rs2412344 and rs183484, and *RRM2* rs1130609) were finally selected (Table 1, Fig. 2).

**Table 1:** Candidate SNPs selected in the study and influence on gene expression according to eQTL database

SNP	Gene	Position	MAF	Alleles	Region	eQTL *P*
rs7543016	*CMPK1*	1:47333967	0.467	C/G	Gly8Arg	0.3
rs3760468	*NME1*	17:51153130	0.376	A/T	Promoter	2.3e^−21^
rs2302254	*NME1*	17:51153539	0.087	C/T	Promoter	2.7e^−13^
rs3744660	*NME2*	17:51168540	0.162	A/G	Intron	2.5e^−12^
rs6102991	*SAMHD1*	20:36956238	0.386	A/G	Promoter	2.0e^−6^
rs28372906	*SAMHD1*	20:36951753	0.063	C/T	5′-UTR	NA
rs6029941	*SAMHD1*	20:36891027	0.447	A/G	3′-UTR	2.1e^−8^
rs3213150	*E2F1*	20:33687284	0.357	C/T	Intron	NA
rs3213180	*E2F1*	20:33675818	0.286	C/G	3′-UTR	NA
rs2412344	*RRM1*	11:4086354	0.430	C/T	Promoter	1.1e^−6^
rs183484	*RRM1*	11:4119902	0.381	G/T	Arg284Arg	9.4e^−6^
rs1130609	*RRM2*	2:10122793	0.343	G/T	5′-UTR/Ser59Ala	1.2e^−7^

*NA* expression data is not available in the eQTL database

Genomic DNA was extracted from blood samples using Blood DNA Kit II (Omega Bio-Tek, USA) according to the manufacturer’s protocol. The 12 candidate SNPs were genotyped by ionization-time-of-flight mass spectrometry (Sequenom, SanDiego, CA). The genotyping success rates were higher than 96.4% except for the rs7543016 polymorphism (89.8%) by the Massarray system. Genotyping results for the SNPs were confirmed in 10% randomly selected samples by PCR-based sequencing.

### Statistical analysis

Statistical analyses were carried out with the IBM SPSS software (version 22.0; IBM SPSS Inc., Chicago, IL, USA). Comparisons of continuous variables between genotype groups were performed by nonparametric test (Mann–Whitney U test). Fitness of the genotype distribution to Hardy–Weinberg Equilibrium (HWE) was analyzed by using the χ^2^ test. Associations of genotypes with CR or non-CR was analyzed by unconditional logistic regression and indicated by odds ratio (OR) and the 95% confidence interval (CI). Survival probabilities were estimated by the Kaplan–Meier method, and differences between genotypes were evaluated using the log-rank test. Cox proportional hazard models were constructed for RFS and OS, adjusting for potential confounding covariates including gender, age at diagnosis, risk stratification, allo-SCT, WBC count, serum lactate dehydrogenase (LDH) level and bone marrow blast counts at diagnosis as necessary. A stepwise selection method was used to determine the potential confounding covariates. Hazard ratio (HR) was used to estimate association of risk factors with RFS and OS. Three genetic models (dominant, recessive and additive model) were tested for the genotype association analysis. *P *< 0.05 was considered to be significant.

## Results

### Baseline characteristics and the overall CR status of the AML patients

The baseline clinical characteristics of the 361 AML patients were summarized in Table 2. The median age of the patients was 43 [range 16–76] years old. Among the AML patients, 196 were male and 165 were female. According to FAB classification, the patients were classified into 7 subtypes. M2 was the most common subtype (53.5%), followed by M5 (20.8%), and no patient with the M7 or M0 subtype was recruited. The median number of WBC was 14.7 × 10^9^/L, the mean serum level of LDH was 363 U/L. Other clinical information was also shown in Table 2. When stratified by cytogenetics and molecular abnormalities, 71, 187, and 78 patients were classified as low risk, intermediate risk, and high risk, respectively. Information of karyotype and somatic mutations was not available for 25 patients. For the 361 patients received Ara-C based induction therapy, 211 patients (58.4%) received Ara-C + mitoxantrone (MA) or Ara-C + idarubicin (IA) regimens, 47 patients (13.0%) received Ara-C + THP (TA), 52 patients (14.4%) received Ara-C + aclarubicin + G-CSF (CAG) and 31 patients (8.6%) received Ara-C + daunorubicin (DA). A total of 205 patients (56.8%) achieved CR after one or two courses of chemotherapy, and CR could not be evaluated accurately for 36 (10.0%) patients due to insufficiencies in clinical evidence. Sixty-four (17.7%) patients received hematopoietic stem cell transplantation, 178 patients died during the follow-up time, and 7 (1.9%) patients were out of touch after several follow-up. The median and mean follow-up period for OS analysis were 926 and 1235 days, respectively.

**Table 2:** The baseline characteristics of 316 AML patients

Characteristics	Totality, n (%)	Median (range)
Gender	361	
Male	196 (54.3%)	
Female	165 (45.7%)	
Age	361	43 (16–76) years
FAB classification		
M1	19 (5.3%)	
M2	193 (53.5%)	
M4	63 (17.5%)	
M5	75 (20.8%)	
M6	6 (1.7%)	
Undefined	5 (1.4%)	
WBC	356 (98.6%)	14.7 (0.4–426.0), × 10^9^/L
LDH	341 (94.5%)	363 (17–5853), U/L
BM blast %	348 (96.4%)	71% (17–99%)
RBC	356 (98.6%)	2.19 (0.67–4.98), × 10^12^/L
Hemoglobin	356 (98.6%)	72 (27–149), g/L
Platelets	356 (98.6%)	34 (2–546), × 10^9^/L
Neutrophil	356 (98.6%)	2.2 (0.0–340.3), × 10^9^/L
Risk stratifications		
Low risk	71 (19.7%)	
Intermediate risk	187 (51.8%)	
High risk	78 (21.6%)	
Undefined	25 (6.9%)	
*FLT3*-*ITD* mutation		
Positive	42 (11.6%)	
Negative	294 (81.4%)	
Unknown	25 (6.9%)	
*NPM1* mutation		
Positive	67 (18.6%)	
Negative	269 (74.5%)	
Unknown	25 (6.9%)	
*CEBPA* mutation		
Positive	50 (13.9%)	
Negative	286 (79.2%)	
Unknown	25 (6.9%)	
Karyotype		
Normal	255 (70.6%)	
Abnormal	84 (23.3%)	
Unknown	22 (6.1%)	
Allo-HCT		
Yes	64 (17.7%)	
No	297 (82.3%)	
CR after two courses of induction therapy		
Yes	205 (56.8%)	
No	120 (33.2%)	
Not evaluated	36 (10.0%)	
Chemotherapy regimens		
MA	125 (34.6%)	
IA	86 (23.8%)	
TA	47 (13.0%)	
DA	31 (8.6%)	
CAG	52 (14.4%)	
Other regimens	20 (5.5%)	

*FAB* French–Britain–American, *WBC* white blood cell, *LDH* lactate dehydrogenase, *BM* bone marrow, *RBC* red blood cell, *Allo-SCT* allogeneic hematopoietic stem cell transplantation, *CR* complete remission, *THP* pirarubicin, *MA* Ara-C + mitoxantrone, *IA* Ara-C + idarubicin, *TA* Ara-C + THP, *DA* Ara-C + daunorubicin, *CAG* Ara-C + aclarubicin + G-CSF

### Comparison of CR rate among genotypes after Ara-C based induction therapy and results of unconditional logistic regression analysis

Significant differences in CR rates among genotypes for the *NME2* rs3744660 (*P *= 0.047), *E2F1* rs3213150 (*P *= 0.043), and *RRM2* rs1130609 (*P *= 0.042) polymorphisms were observed (*p* values from Chi square test by three genotype groups). For the *NME2* rs3744660 polymorphism, the CR rate was 68.5, 53.9, and 57.1%, respectively, for the GG, GA and AA genotypes, and carriers of the rs3744660 A allele (dominant model) showed significantly increased non-CR (indicative of chemoresistance) rate (GA+AA vs GG, 45.6% vs 31.5%, crude OR = 1.829, 95% CI 1.127–2.967, *P *= 0.014) (Table 3). For the *E2F1* rs3213150 polymorphism, the CR rate was 60.4, 69.2, and 50.0%, respectively, for the CC, CT, and TT genotypes, and rs3213150 TT homozygous showed significantly increased non-CR rate as compared with carriers of the rs3213150 C allele (recessive model) (50.0% vs 34.4%, crude OR = 1.903, 95% CI 1.014–3.574, *P *= 0.043). For the *RRM2* rs1130609 polymorphism, the CR rate was 64.1, 67.1, and 44.1%, respectively, for the TT, TG and GG genotypes, and patients with the rare GG genotype showed significantly increased non-CR rate as compared with carriers of the T allele (GG vs TT+TG, 55.9% vs 34.3%, crude OR = 2.428, 95% CI 1.181–4.990, *P *= 0.014). Carriers of the *SAMHD1* rs6102991 G allele (GA+GG) also showed marginally decreased non-CR rate as compared with the wild-type AA homozygotes (crude OR = 0.611, 95% CI 0.369–1.013, *P *= 0.055). No differences in CR rates among genotypes for other SNPs were observed (Table 3).

**Table 3:** Comparison of CR rate among genotypes after 2 courses of Ara-C based chemotherapy

Genotype	Total (n)	CR, n (%)	Non-CR, n (%)	*P*	OR (95% CI)	*P* ^a^	OR (95% CI)^a^
*CMPK1* rs7543016							
CC	93	57 (61.3%)	36 (38.7%)		1.00 (reference)		1.00 (reference)
GC	167	106 (63.5%)	61 (36.5%)	0.727	0.911 (0.540–1.537)	0.921	0.970 (0.531–1.772)
GG	30	23 (76.7%)	7 (23.3%)	0.125	0.482 (0.188–1.238)	0.076	0.365 (0.120–1.111)
*NME1* rs3760468							
AA	115	77 (67.0%)	38 (33.0%)		1.00 (reference)		1.00 (reference)
AT	154	96 (62.3%)	58 (37.7%)	0.434	1.224 (0.737–2.033)	0.256	1.420 (0.775–2.597)
TT	44	26 (59.1%)	18 (40.9%)	0.353	1.403 (0.686–2.869)	0.465	1.359 (0.597–3.096)
*NME1* rs2302254							
CC	186	123 (66.1%)	63 (33.9%)		1.00 (reference)		1.00 (reference)
CT	114	65 (57.0%)	49 (43.0%)	0.114	1.473 (0.912–2.375)	0.288	1.355 (0.775–2.370)
TT	19	13 (68.4%)	6 (31.6%)	0.840	0.901 (0.327–2.481)	0.534	0.688 (0.212–2.232)
*NME2* rs3744660							
GG	213	146 (68.5%)	67 (31.5%)		1.00 (reference)		1.00 (reference)
AG	89	48 (53.9%)	41 (46.1%)	0.016	1.861 (1.121–3.091)	0.024	1.953 (1.092–3.484)
AA	14	8 (57.1%)	6 (42.9%)	0.376	1.634 (0.545–4.897)	0.770	1.299 (0.361–4.673)
AA+AG	103	56 (54.4%)	47 (45.6%)	0.014	1.829 (1.127–2.967)	0.030	1.852 (1.062–3.236)
*SAMHD1* rs6102991							
AA	88	49 (55.7%)	39 (44.3%)		1.00 (reference)		1.00 (reference)
GA	161	107 (66.5%)	54 (33.5%)	0.093	0.634 (0.372–1.080)	0.183	0.669 (0.370–1.209)
GG	62	43 (69.4%)	19 (30.6%)	0.090	0.555 (0.280–1.101)	0.063	0.483 (0.224–1.038)
AG+GG	223	150 (67.3%)	73 (32.7%)	0.055	0.611 (0.369–1.013)	0.086	0.611 (0.349–1.071)
*SAMHD1* rs28372906							
TT	284	180 (63.4%)	104 (36.6%)		1.00 (reference)		1.00 (reference)
CT	32	21 (65.6%)	11 (34.4%)	0.802	0.907 (0.420–1.955)	0.878	0.935 (0.394–2.217)
CC	4	3 (75.0%)	1 (25.0%)	0.632	0.577 (0.059–5.618)	0.953	0.933 (0.091–9.524)
*SAMHD1* rs6029941							
GG	88	50 (56.8%)	38 (43.2%)		1.00 (reference)		1.00 (reference)
GA	161	107 (66.5%)	54 (33.5%)	0.132	0.664 (0.389–1.132)	0.339	0.739 (0.398–1.374)
AA	65	42 (64.6%)	23 (35.4%)	0.330	0.721 (0.372–1.395)	0.370	0.706 (0.330–1.512)
*E2F1* rs3213180							
GG	149	95 (63.8%)	54 (36.2%)		1.00 (reference)		1.00 (reference)
GC	141	90 (63.8%)	51 (36.2%)	0.990	0.997 (0.617–1.610)	0.915	0.970 (0.552–1.704)
CC	29	16 (55.2%)	13 (44.8%)	0.383	1.429 (0.639–3.195)	0.429	1.449 (0.578–3.636)
*E2F1* rs3213150							
CC	111	67 (60.4%)	44 (39.6%)		1.00 (reference)		1.00 (reference)
CT	159	110 (69.2%)	49 (30.8%)	0.133	0.678 (0.408–1.127)	0.105	0.616 (0.343–1.106)
TT	46	23 (50.0%)	23 (50.0%)	0.232	1.523 (0.762–3.042)	0.128	1.835 (0.839–4.016)
CC+CT	270	177 (65.6%)	93 (34.4%)		1.00 (reference)		1.00 (reference)
TT	46	23 (50.0%)	23 (50.0%)	0.043	1.903 (1.014–3.574)	0.033	2.208 (1.065–4.567)
*RRM1* rs183484							
GG	100	62 (62.0%)	38 (38.0%)		1.00 (reference)		1.00 (reference)
GT	177	118 (66.7%)	59 (33.3%)	0.434	0.816 (0.490–1.359)	0.112	0.617 (0.340–1.119)
TT	42	22 (52.4%)	20 (47.6%)	0.287	1.483 (0.716–3.071)	0.263	1.616 (0.697–3.731)
*RRM1* rs2412344							
TT	89	56 (62.9%)	33 (37.1%)		1.00 (reference)		1.00 (reference)
CT	183	119 (65.0%)	64 (35.0%)	0.734	0.913 (0.539–1.545)	0.510	0.817 (0.449–1.488)
CC	48	28 (58.3%)	20 (41.7%)	0.599	1.212 (0.592–2.483)	0.967	1.018 (0.429–2.415)
*RRM2* rs1130609							
TT	131	84 (64.1%)	47 (35.9%)		1.00 (reference)		1.00 (reference)
GT	149	100 (67.1%)	49 (32.9%)	0.599	0.876 (0.534–1.436)	0.920	0.972 (0.557–1.697)
GG	34	15 (44.1%)	19 (55.9%)	0.034	2.264 (1.053–4.867)	0.069	2.347 (0.935–5.882)
TT+GT	280	184 (65.7%)	96 (34.3%)		1.00 (reference)		1.00 (reference)
GG	34	15 (44.1%)	19 (55.9%)	0.014	2.428 (1.181–4.990)	0.044	2.398 (1.025–5.618)

^a^Adjusted by age, risk stratification, WBC count, and serum LDH level

Logistic regression analysis showed significant associations of risk stratification, age, pretreatment WBC counts and LDH levels with non-CR risk (Additional file 1: Table S1). When adjusted by these risk factors, *NME2* rs3744660, *E2F1* rs3213150 and *RRM2* rs1130609 were still significantly associated with risk of non-CR (Table 3). *NME2* rs3744660 AG genotype or carriers of the rs3744660 A allele showed increased risk for non-CR (AG vs GG: adjusted OR = 1.953, 95% CI 1.092–3.484, *P *= 0.024; AG+AA vs GG: adjusted OR = 1.852, 95% CI 1.062–3.236, *P *= 0.030). For the *E2F1* rs3213150 polymorphism, as compared with patients carrying the *E2F1* rs3213150 C allele, those with the TT genotype showed increased risk of non-CR (adjusted OR = 2.208, 95% CI 1.065–4.567, *P *= 0.033). While for the *RRM2* rs1130609 polymorphism, the rare GG homozygotes showed significantly increased risk of non-CR in comparison with carriers of the T allele (adjusted OR = 2.398, 95% CI 1.025–5.618, *P *= 0.044). Marginally decreased risk of non-CR was also observed for the *SAMHD1* rs6102991 polymorphism in a dominant model (GA+GG vs AA, adjusted OR = 0.611, 95% CI 0.349–1.071, *P *= 0.086).

### Combined influence of *E2F1* and *RRM2* polymorphisms on chemosensitivity to Ara-C based induction therapy in AML patients

As E2F1 is a transcription factor involved in the regulation of *RRM2* expression, a combined genotype analysis of the *E2F1* and *RRM2* polymorphisms on CR rate was carried out. As both *E2F1* rs3213150 and *RRM2* rs1130609 polymorphisms were associated with increased risk for non-CR in negative models, patients were grouped based on genotypes of the two SNPs (Table 5): rs1130609 TT+GT/rs3213150 CC+CT (both favorable for CR, n = 237), rs1130609 TT+GT/rs3213150 TT, n = 37), rs1130609 GG/rs3213150 CC+CT (n = 27), and rs1130609 GG/rs3213150 TT (both unfavorable for CR, n = 6). Significant difference in CR rate was observed among the four groups (*χ*^2^ = 10.637, *P *= 0.014), which was 66.7, 54.1, 48.1, and 16.7%, respectively (Table 5). As compared with the favorable genotypes combination group (rs1130609 TT+GT/rs3213150 CC+CT), carriers of two unfavorable genotypes (rs1130609 GG/rs3213150 TT) and those carrying at least one unfavorable genotype showed significantly increased risk of non-CR (OR = 10.00, 95% CI 1.149–87.06, *P *= 0.004; OR = 2.118, 95 CI 1.233–3.637, *P *= 0.006, respectively, Table 4). After adjustment for age, risk stratification, and WBC counts, results of logistic regression analysis indicated that patients carrying both or at least one unfavorable genotype showed significantly increased risk of non-CR (OR = 9.780, 95% CI 1.099–87.069, *P *= 0.041; OR = 2.257, 95 CI 1.273–4.002, *P *= 0.005, respectively, Table 4).

**Table 4:** Combined genotypes of *RRM2* and *E2F1* polymorphisms and chemotherapy sensitivity in AML patients

rs1130609	rs3213150	CR, n (%)	Non-CR, n (%)	*P*	OR (95% CI)	*P* ^b^	OR (95% CI)^b^
TT+GT	CC+CT	158 (66.7%)	79 (33.3%)		1.00 (reference)		1.00 (reference)
TT+GT	TT	20 (54.1%)	17 (45.9%)	0.134	1.700 (0.844–3.426)	0.048	2.115 (1.006–4.450)
GG	CC+CT	13 (48.1%)	14 (51.9%)	0.056	2.154 (0.966–4.802)	0.159	1.840 (0.787–4.301)
GG	TT	1 (16.7%)	5 (83.3%)	0.004	10.00 (1.149–87.06)	0.041	9.780 (1.099–87.069)
Unfavorable combinations^a^		34 (48.6%)	36 (51.4%)	0.006	2.118 (1.233–3.637)	0.005	2.257 (1.273–4.002)

^a^Unfavorable combination genotypes included rs1130609 GG genotype or rs3213150 TT genotype

^b^Adjusted by age, risk stratification, WBC count, and serum LDH level

### Influence of candidate SNPs on RFS and OS for AML patients

**Fig. 3:**
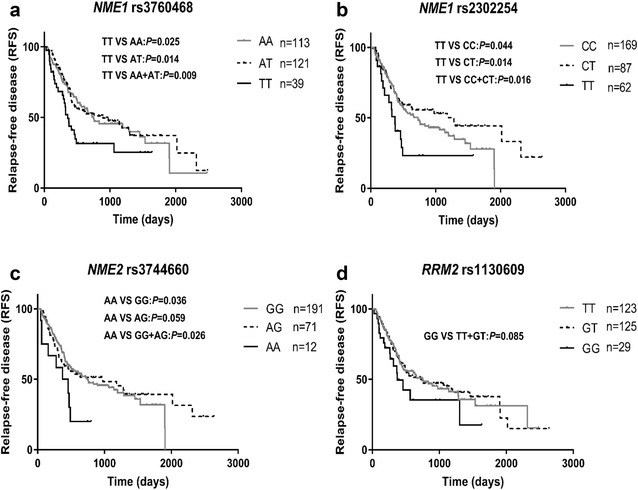
Comparison of relapse-free survival (RFS) in AML patients among/between genotypes of *NME1* rs3760468 (**a**); *NME1* rs2302254 (**b**); *NME2* rs3744660 (**c**); and *RRM2* rs1130609 (**d**)

Univariate analysis showed significant differences in RFS among genotypes of *NME1* rs3760468, *NME1* rs2302254, and *NME2* rs3744660 (Table 5, Fig. 3). All the three SNPs were associated with worse RFS in a recessive model (rs3760468 TT vs AA+AT: HR = 1.752, 95% CI 1.142–2.686, *P *= 0.009; rs2302254 TT vs CC+CT: HR = 1.912, 95% CI 1.117–3.272, *P *= 0.016; rs3744660 AA vs GG+AG: HR = 2.087, 95% CI 1.051–4.145, *P *= 0.036). The *RRM2* rs1130609 GG genotype also showed marginally worse RFS (GG vs TT+GT: HR = 1.577, 95% CI 0.935–2.660, *P *= 0.085).

**Table 5:** Univariate and multivariate Cox regression analysis of SNPs associated with AML relapse-free survival (RFS)

Genotype	n	Mean ± SE (day)	Median (range, day)	HR (95% CI)	*P*	HR (95% CI)^a^	*P* ^a^
*NME1* rs3760468							
AA	113	1058 ± 202	751 (294–1208)	1.00 (reference)		1.00 (reference)	
AT	121	1163 ± 212	894 (378–1410)	0.946 (0.657–1.362)	0.764	1.027 (0.709–1.488)	0.887
TT	39	655 ± 207	357 (285–429)	1.703 (1.070–2.709)	0.025	1.744 (1.085–2.801)	0.022
AA+AT	234	1110 ± 149	761 (379–1143)	1.00 (reference)		1.00 (reference)	
TT	39	655 ± 207	357 (285–429)	1.752 (1.142–2.686)	0.009	1.854 (1.197–2.871)	0.006
*NME1* rs2302254							
CC	169	941 ± 129	724 (4484–964)	1.00 (reference)		1.00 (reference)	
CT	87	1324 ± 266	1282 (516–2048)	0.770 (0.526–1.126)	0.177	0.728 (0.494–1.071)	0.107
TT	22	581 ± 253	373 (286–460)	1.756 (1.014–3.042)	0.044	1.688 (0.973–2.928)	0.062
CC+CT	256	1111 ± 156	760 (398–1122)	1.00 (reference)		1.00 (reference)	
TT	22	581 ± 253	373 (286–460)	1.912 (1.117–3.272)	0.016	1.878 (1.096–3.217)	0.022
*NME2* rs3744660							
GG	191	987 ± 124	751 (397–1105)	1.00 (reference)		1.00 (reference)	
AG	71	1239 ± 283	976 (186–1766)	0.929 (0.627–1.377)	0.714	0.958 (0.644–1.425)	0.832
AA	12	382 ± 116	373 (95–651)	2.087 (1.051–4.145)	0.036	2.224 (1.114–4.444)	0.024
GG+AG	262	1136 ± 157	760 (399–1121)	1.00 (reference)		1.00 (reference)	
AA	12	382 ± 116	373 (95–651)	2.127 (1.078–4.194)	0.026	2.250 (1.135–4.460)	0.020
*RRM2* rs1130609							
TT	123	1126 ± 208	751 (368–1134)	1.00 (reference)		1.00 (reference)	
GT	125	1129 ± 216	724 (209–1239)	1.561 (0.692–1.386)	0.907	0.926 (0.651–1.317)	0.668
GG	29	679 ± 272	371 (200–542)	1.561 (0.900–2.707)	0.113	1.437 (0.825–2.501)	0.200
TT+GT	248	1127 ± 159	729 (381–1077)	1.00 (reference)		1.00 (reference)	
GG	29	679 ± 272	371 (200–542)	1.577 (0.935–2.660)	0.085	1.493 (0.882–2.528)	0.136

^a^Adjusted for risk stratification, WBC count, and allo-SCT

Multivariate analysis indicated that risk stratification, age, allo-SCT, WBC counts and serum LDH levels were associated with RFS of AML patients (Additional file 2: Table S2). When adjusted by these risk factors, *NME1* rs3760468 TT, *NME1* rs2302254 TT, and *NME2* rs3744660 GG genotypes were still associated with worse RFS (rs3760468 TT vs AA+AT: adjusted HR = 1.854, 95% CI 1.197–2.871, *P *= 0.006; rs2302254 TT vs CC+CT: adjusted HR = 1.878, 95% CI 1.096–3.217, *P *= 0.022; rs3744660 AA vs GG+AG: adjusted HR = 2.250, 95% CI 1.135–4.460, *P *= 0.020).

**Fig. 4:**
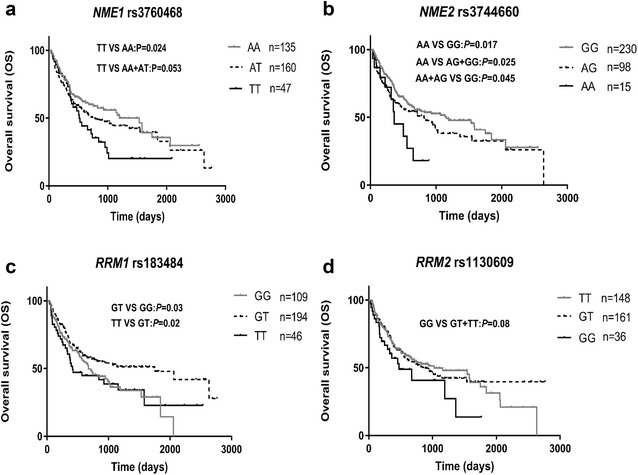
Comparison of overall survival (OS) in AML patients among/between genotypes of *NME1* rs3760468 (**a**); *NME2* rs3744660 (**b**); *RRM1* rs183484 (**c**); and *RRM2* rs1130609 (**d**)

Results of univariate analysis showed that the *NME1* rs3760468, *NME2* rs3744660, and *RRM1* rs183484 polymorphisms were associated with OS of AML patients significantly (Table 6, Fig 4). As compared with rs3760468 AA homozygotes, rs3760468 TT homozygotes showed significantly worse OS (TT vs AA, HR = 1.652, 95% CI 1.067–2.557, *P *= 0.024) and carriers of the rs3760468 T allele showed a trend of worse OS (TT+AT vs AA: HR = 1.328, 95% CI 0.973–1.812, *P *= 0.073). For the rs3744660 polymorphisms, the rare AA homozygotes showed significantly worse OS (HR = 2.214, 95% CI 1.154–4.247, *P *= 0.017). For the *RRM1* rs183484 polymorphism, the GT heterozygotes showed significantly better OS than the wild-type GG homozygotes (HR = 0.692, 95% CI 0.496–0.964, *P *= 0.030). Marginally significant difference in OS between the *RRM2* rs1130609 GG and GT+TT genotypes was also observed (HR = 1.516, 95% CI 0.948–2.422, *P *= 0.080, Table 6).

**Table 6:** Univariate and multivariate Cox regression analysis of SNPs associated with AML overall survival (OS)

Genotype	n	Mean ± SE (day)	Median (range) (day)	HR (95% CI)	*P*	HR (95% CI)^a^	*P* ^a^
*NME1* rs3760468							
AA	135	1326 ± 209	1534 (947–2121)	1.00 (reference)		1.00 (reference)	
AT	160	1232 ± 218	768 (388–1148)	1.238 (0.890–1.724)	0.205	1.455 (1.015–2.087)	0.041
TT	47	801 ± 231	513 (398–638)	1.652 (1.067–2.557)	0.024	1.702 (1.072–2.704)	0.024
AT+TT	207	1159 ± 185	663 (442–884)	1.328 (0.973–1.812)	0.073	1.518 (1.082–2.129)	0.016
*NME2* rs3744660							
GG	230	1283 ± 168	1159 (615–1703)	1.00 (reference)		1.00 (reference)	
AG	98	1175 ± 252	817 (410–1224)	1.283 (0.924–1.780)	0.137	1.181 (0.831–1.679)	0.353
AA	15	442 ± 149	359 (167–551)	2.214 (1.154–4.247)	0.017	2.284 (1.145–4.559)	0.019
GG+AG	328	1261 ± 143	988 (682–1294)	1.00 (reference)		1.00 (reference)	
AA	15	442 ± 149	359 (167–511)	2.054 (1.080–3.905)	0.025	2.172 (1.098–4.294)	0.026
*RRM1* rs183484							
GG	109	940 ± 167	672 (454–890)	1.00 (reference)		1.00 (reference)	
GT	194	1506 ± 202	1749 (799–2699)	0.692 (0.496–0.964)	0.030	0.599 (0.413–0.868)	0.007
TT	46	993 ± 305	414 (162–666)	1.112 (0.717–1.726)	0.635	1.063 (0.665–1.698)	0.799
*RRM2* rs1130609							
TT	148	1243 \± 201	1025 (426–1624)	1.00 (reference)		1.00 (reference)	
GT	161	1380 ± 209	843 (520–1166)	0.988 (0.719–1.357)	0.941	1.025 (0.746–1.408)	0.881
GG	36	753 ± 248	473 (137–809)	1.506 (0.917–2.475)	0.106	1.489 (0.906–2.448)	0.116
TT+GT	309	1297 ± 153	935 (645–1225)	1.00 (reference)		1.00 (reference)	
GG	36	753 ± 248	473 (137–809)	1.516 (0.948–2.422)	0.080	1.471 (0.920–2.352)	0.107

^a^Adjusted for risk stratification, WBC count, allo-SCT, age, and serum LDH level

Results from multivariate analysis indicated that risk stratification, age, allo-SCT, WBC counts and serum LDH levels were associated with OS of AML patients (Additional file 2: Table S2). When adjusted by these OS risk factors, rs3760468, rs3744660, and rs183484 polymorphisms were still associated with OS risk. As compared with rs3760468 AA homozygotes, rs3760468 TT homozygotes or carriers of the rs3760468 T allele showed significantly worse OS (TT+AT vs AA: adjusted HR = 1.518, 95% CI 1.082–2.129, *P *= 0.016). For the rs3744660 polymorphisms, the rare AA homozygotes showed significantly worse OS (adjusted HR = 2.284, 95% CI 1.145–4.559, *P *= 0.019). For the *RRM1* rs183484 polymorphism, the GT heterozygotes showed significantly longer OS than the wild-type GG homozygotes (HR = 0.599, 95% CI 0.413–0.868, *P *= 0.007).

## Discussion

In this study, we observed the associations of 12 SNPs in 7 genes involved in Ara-C and dNTP metabolic pathway with chemosensitivity to Ara-C based therapy as well as disease prognosis in Chinese AML patients. We observed that 3 SNPs including *NME2* rs3744660 (dominant model), *E2F1* rs3213150 (recessive model) and *RRM2* rs1130609 (recessive model) were associated with increased risk for non-CR after Ara-C based therapy, while the *SAMHD1* rs6102991 polymorphism showed a tendency with decreased risk of non-CR. We also observed a combined effect of *E2F1* and *RRM2* polymorphisms on CR rate. In addition, we observed that the *NME1* rs3760468 and rs2302254, and *NME2* rs3744660 polymorphisms predicted worse RFS, while *NME1* rs3760468, *NME2* rs3744660, and *RRM1* rs183484 predicted worse OS in the AML patients.

For the 7 genes involved in our study, 3 genes (*CMPK1*, *NME1* and *NME2*) were involved in the metabolic activation of Ara-C, while 4 genes (*SAMHD1*, *E2F1*, *RRM1* and *RRM2*) were involved in the Ara-C inactivation or DCK feedback inhibition (dNTP/dCTP synthesis) pathways. Four genes (*CMPK1*, *NME2, SAMHD1* and *E2F1*) were studied for the first time in our study. The majority of the SNPs included in our study were eQTL variants. For the 4 SNPs associated with CR rate observed in our study, difference in mRNA expression among genotypes of *NME2* rs3744660 (*P *= 2.5e^−12^), *RRM2* rs1130609 (*P *= 1.2e^−7^), and *SAMHD1* rs6102991 (*P *= 2.0e^−6^) were indicated by eQTL analysis, and 2 of these SNPs (*NME2* rs3744660, *SAMHD1* rs6102991) were reported for the first time in our study.

*NME1* (alias DNPK-A) and *NME2* (alias DNPK-B) are two most important members of the NDPK family of proteins and mediate phosphorylation of ara-CDP into ara-CTP. The coding genes *NME1* and *NME2* are closely neighbored on chromosome 17. We observed that carriers of the *NME2* intronic variant rs3744660 was associated with decreased *NME2* mRNA expression shown by eQTL analysis, which indicated decreased DNPK activity and ara-CTP formation, this is in agreement with the observation of decreased CR rate in patients carrying the rs3744660 A allele or worse prognosis in AA genotyped patients. Few studies have focused on *NME1*–*NME2* locus presently. *NME1* is initially identified for its metastatic suppressive potential for cancer cells [28]. Qu et al. reported that the *NME1* promoter SNPs rs2302254 and rs3760468 can alter nuclear proteins binding capacity and reduce *NME1* promoter activity by about 20%, which may account for increased breast cancer mortality for these SNPs in Chinese [29]. In a study by Braunagel et al. the rs2302254 TT genotype (mutant homozygotes) was an independent risk factor for Ara-C associated neurotoxicity but not for OS or RFS in AML patients [26]. However, neither rs2302254 nor rs3760468 was associated with CR rate in our study, though both polymorphisms can results in decreased *NME1* mRNA expression and predicted worse prognosis indicated by RFS and/or OS in our study. We assume that the positive findings in AML prognosis could also be explained, at least partially, by the direct functions of *NME1/NME2*, both can stimulate the growth and survival of AML cells through activation of the MAPK and transducers and activators of transcription signaling pathways [30, 31]. Of note, the rs3744660 is located in intron 4 of *NME2* (*NM*_001018137.2) and intron 7 of the *NME1*–*NME2* transcript (*NM*_001018136.2), the exact function of this SNP remains to be explored.

The *RRM2* rs1130609 polymorphism is a non-synonymous SNP that leads to amino acid change from Ser59 to Ala59. We found that the rare GG genotype showed increased *RRM2* mRNA expression as indicated by eQTL analysis, which may suggest increased dNTP synthesis and increased feedback inhibition on *DCK* activity in these patients. This is in accordance with the findings of increased non-CR risk and marginally worse prognosis in AML patients with the GG genotype in our study. In consistent with our study, higher *RRM1*/*RRM2* mRNA expression was observed to be associated with decreased Ara-C sensitivity in the HapMap YRI samples (30 trios). Our findings are also in agreement with a previous study by Cao et al. who reported that the rs1130609 polymorphism was associated with decreased Ara-C cytotoxicity in the HapMap CEU samples and worse EFS in Caucasian AML patient cohorts [18]. The exact function of the SNP remains unknown despite its influence on *RRM2* mRNA expression. Search of the dbSNP database shows that the rs1130609 polymorphism is also a 5′-UTR SNP for the *RRM2* transcript NM_001034.3. Further functional study of this SNP is required. Cao et al. reported significant associations of several *RRM1* SNPs in the regulatory regions (e.g. rs2412344, rs11030907, rs7929397, rs2898950, rs1042919, and rs2412344) with ex vivo Ara-C cytotoxicity and/or in vivo Ara-C response [18]. A recent study in Chinese AML patients also observed association of the 3′-UTR potential miRNA binding site SNP rs1042919 with decreased CR rate but not disease prognosis in AML patients [32]. However, we failed to observed associations between the two *RRM1* SNPs rs2412344 (in promoter) and rs183484 (Arg284Arg) and risk of non-CR, though significant differences in mRNA expression among the genotypes of both SNPs were indicated by eQTL analysis. We observed that the rs183484 GT genotype showed better OS (HR = 0.599, 95% CI 0.413–0.868, *P *= 0.007). The discrepancies among the studies should be further validated in future studies.

*E2F1* is a newly identified risk gene for Ara-C response in our study. We selected this gene based on a recent report of ERK/E2F1 signaling in regulating *RRM2* expression, dCTP pool and gemcitabine sensitivity [17]. The *E2F1* rs3213150 polymorphism is an intronic and tag SNP selected in our study. Interesting, we observed significant association of this SNP with CR rate after Ara-C based induction therapy. In addition, a combined effect of the *E2F1* rs3213150 and *RRM2* rs1130609 polymorphisms on CR rate is observed. The CR rate decreased with the number of unfavorable genotypes carried at both loci (66.7% in carriers of both favorable genotypes, 51.6% in carriers of one unfavorable genotype, and 16.6% in carriers of unfavorable genotypes for both SNPs), and unfavorable genotypes at both loci results in about ninefold increase in non-CR risk after Ara-C based chemotherapy (OR = 9.780, 95% CI 1.099–87.069, *P *= 0.041). Our study suggest the importance of concomitant consideration of genetic variants of both *E2F1* and *RRM2* in evaluation of Ara-C response in AML patients.

*SAMHD1* is a recently identified ara-CTPase that can hydrolyze and detoxify intracellular ara-CTP [12]. Inhibition of the enzyme or CRISPR–Cas9-mediated disruption of *SAMHD1* can sensitize AML cells to Ara-C [13]. In our study, we analyzed associations of three *SAMHD1* SNPs (rs6102991 in the promoter, rs28372906 in 5′-UTR, and rs6029941 in 3′-UTR) with drug response and AML prognosis. We observed that carriers of the rs6102991 G allele showed a tendency with decreased risk of non-CR after Ara-C based therapy in a dominant model (OR = 0.611, 95% CI 0.369–1.013, *P *= 0.055). The rs6102991 polymorphism is located at − 4395 upstream transcription start site (T-4395C). eQTL analysis indicates that the rs6102991 polymorphism results in decreased *SAMHD1* mRNA expression, which supports our clinic findings. As the SNPs is far from the transcription start site, our findings indicate potential influence of rs6102991 or other variants in high LD with it on Ara-C response through affecting *SAMHD1* mRNA expression. Few studies have focused on functions of rare *SAMHD1* coding variations on HIV infection and replication [33, 34], however, there is still a gap between *SAMHD1* variations and the clinical relevance. Because SNPs information for the *SAMHD1* promoter is very limited in the SNP database available, further study is required to explore the potentially causative variant(s).

There were some limitations in our study. For instance, the outcomes of our study failed to undergo multiple test adjusting, some *P* values lost statistical significance when Bonferroni correction was performed possibly due to limited sample size included in our study. In addition, Ara-C response is affected by various genetic factors, the contribution of any unique gene in the drug response might be limited. And due to the limitations of the Massarray genotyping system, we failed to genotype some of the important SNPs such as *RRM1*rs1561876 [18]. For the AML patients involved in our study, genotype distribution for 4 SNPs (*CMPK1* rs7543016, *SAMHD1* rs28372906, *RRM1* rs183484, and *RRM2* rs1130609) were not in Hardy–Weinberg equilibrium, these may partially be explained by the internal function of the genes in etiology of AML rather than in Ara-c response. We suppose that these polymorphisms may also act as susceptibility genes for AML, which may account for the abnormal distribution of the genotype for these SNPs in the AML patients. Of course, this should be confirmed in further study.

## Conclusions

Our current study demonstrated that SNPs in genes involved in Ara-C metabolic pathway are associated with drug response to Ara-C based induction therapy and prognosis of AML in Chinese patients, though further confirmatory studies and functional evaluations of the newly identified SNPs are needed to further support our findings. In addition, we suggest consideration of combined genotypes in the Ara-C metabolic pathway in evaluation of the associations between genetic variations and drug response in AML. Our findings provide insightful information for the understanding of individual difference in drug response and potential biomarkers for identification of patients with increased risk of chemoresistance or poor prognosis.

## Additional files

**Additional file 1: Table S1.** Unconditional logistic regression analysis of clinical variables associated with non-CR risk in AML patients.

**Additional file 2: Table S2.** Multivariate Cox regression analysis of clinical factors influencing AML OS and RFS.

## Abbreviations

Ara-C: cytarabine arabinoside
dNTP: deoxy-ribonucleoside triphosphate
AML: acute myeloid leukemia
ara-CTP: cytarabine arabinoside triphosphate
CR: complete remission
RFS: relapse-free survival
OS: overall survival
eQTL: expression Quantitative Trait Loci
SNPs: single nucleotide polymorphisms
*SLC29A1*: solute carrier family 29 member 1
*DCK*: deoxycytidine kinase
NDPKs: nucleoside diphosphate kinases
dCTP: deoxycytidine triphosphate
*SAMHD1*: SAM domain and HD domain 1
dATP: deoxyadenosine triphosphate
dTTP: deoxythymidine triphosphate
dGTP: deoxyguanosine triphosphate
RR: ribonucleotide reductase
CTP: cytidine triphosphate
*RRM1*: ribonucleotide reductase catalytic subunit M1
*RRM2*: ribonucleotide reductase catalytic subunit M2
*WT1*: Wilms tumor 1
*DNMT3A*: DNA methyltransferase 3 alpha
*E2F1*: transcription factor 1
*FLT3*: FMS-like Tyrosine Kinase3
*NMP1*: nucleophosmin
*NME1*: nucleoside diphosphate kinase 1
*NME2*: nucleoside diphosphate kinase 2
EFS: event free survival
*CMPK1*: cytidine/uridine monophosphate kinase 1
EMR: electronic medical record
HSCT: haematopoietic stem cell transplantation
HWE: Hardy–Weinberg Equilibrium
FAB: French–Britain–American
WBC: white blood cell
LDH: lactate dehydrogenase
BM: bone marrow
RBC: red blood cell
Allo-SCT: allogeneic hematopoietic stem cell transplantation
CR: complete remission
THP: pirarubicin
MA: Ara-C + mitoxantrone
IA: Ara-C + idarubicin
TA: Ara-C + pirarubicin
DA: Ara-C + daunorubicin
CAG: Ara-C + aclarubicin + G-CSF
G-CSF: granulocyte colony-stimulating factor
OR: odds ratio
HR: hazard ratio
CI: confidence interval
LD: linkage disequilibrium
HIV: human immunodeficiency virus
