# Supplementary material for: Association of genetic polymorphisms in genes involved in Ara-C and dNTP metabolism pathway with chemosensitivity and prognosis of adult acute myeloid leukemia (AML)
Source: J Transl Med. 2018 Apr 10;16:90. doi: 10.1186/s12967-018-1463-1 (PMC5892020; doi:10.1186/s12967-018-1463-1)
Supplement: Supplementary file 1 — Additional file 1: Table S1. Unconditional logistic regression analysis of clinical variables associated with non-CR risk in AML patients. [file 12967_2018_1463_MOESM1_ESM.docx]

**Table S1. Unconditional logistic regression analysis of clinical variables associated with non-CR risk in AML patients.**

| **Variables in the model** | ***P*** | **OR (95%CI)** |
| --- | --- | --- |
| WBC | 0.004 | 0.987 (0.979-0.996) |
| Risk stratification groups: |  |  |
| Low *vs* intermediate | 0.040 | 0.439 (0.200-0.965) |
| High *vs* intermediate | 0.119 | 1.689 (0.875-3.268) |
| LDH | 0.017 | 0.999 (0.999-1.000) |
| Age | 0.023 | 0.978 (0.959-0.997) |
| Neutrophil | 0.096 | 1.011 (0.998-1.024) |
| Gender | 0.673 | 0.888 (0.510-1.544) |
| BM blasts percentage | 0.222 | 2.444 (0.5822-10.256) |
| RBC | 0.850 | 0.922 (0.395-2.152) |
| Hemoglobin | 0.374 | 1.014 (0.983-1.046) |
| Platelets | 0.262 | 0.998 (0.994-1.002) |

**Abbreviation**: WBC, white blood cell; Allo-SCT, allogeneic hematopoietic stem cell transplantation; LDH, lactate dehydrogenase. BM, bone marrow; RBC,red blood cell.
