# Supplementary material for: Association of genetic polymorphisms in genes involved in Ara-C and dNTP metabolism pathway with chemosensitivity and prognosis of adult acute myeloid leukemia (AML)
Source: J Transl Med. 2018 Apr 10;16:90. doi: 10.1186/s12967-018-1463-1 (PMC5892020; doi:10.1186/s12967-018-1463-1)
Supplement: Supplementary file 2 — Additional file 2: Table S2. Multivariate Cox regression analysis of clinical factors influencing AML OS and RFS. [file 12967_2018_1463_MOESM2_ESM.docx]

**Table S2**. **Multivariate Cox regression analysis of clinical factors influencing AML OS and RFS.**

| **Clinical factors** | **Influence on OS** | | | |  | | **Influence on RFS** | | | |
| --- | --- | --- | --- | --- | --- | --- | --- | --- | --- | --- |
|  | ***P*** | | **HR (95%CI)** | |  | | ***P*** | | **HR (95%CI)** | |
| WBC | 1.6×10^-4^ | 1.01 (1.00-1.00) | |  | | 0.006 | | 1.01 (1.00-1.01) | |  |
| Risk stratification: |  |  | |  | |  | |  | |  |
| Low vs intermediate | 0.002 | 0.44 (0.26-0.74) | |  | | 0.104 | | 0.67 (0.42-1.08) | |  |
| High vs intermediate | 0.001 | 1.96 (1.33-2.87) | |  | | 0.007 | | 1.83 (1.18-2.84) | |  |
| Allo-SCT: Yes vs No | <1×10^-6^ | 0.15 (0.07-0.31) | |  | | 1.8×10^-5^ | | 0.28 (0.16-0.50) | |  |
| LDH | 5×10^-6^ | 1.00 (1.00-1.01) | |  | | 0.390 | | 1.00 (1.00-1.01) | |  |
| Age | 0.006 | 1.02 (1.01-1.03) | |  | | 0.181 | | 1.01 (1.00-1.02) | |  |
| Gender | 0.108 | 1.32 (0.94-1.86) | |  | | 0.322 | | 1.22 (0.82-1.81) | |  |
| BM blasts percentage | 0.464 | 1.01 (0.99-1.03) | |  | | 0.484 | | 1.01 (0.99-1.03) | |  |
| Neutrophil | 0.951 | 1.00 (0.99-1.01) | |  | | 0.449 | | 1.00 (0.99-1.01) | |  |
| RBC | 0.482 | 0.84 (0.52-1.36) | |  | | 0.734 | | 0.91 (0.508-1.61) | |  |
| Hemoglobin | 0.312 | 0.99 (0.97-1.01) | |  | | 0.677 | | 1.00 (0.98-1.02) | |  |
| Platelets | 0.304 | 1.00 (0.99-1.01) | |  | | 0.583 | | 1.00 (1.00-1.01) | |  |

**Abbreviation**: WBC, white blood cell; Allo-SCT, allogeneic hematopoietic stem cell transplantation;LDH, lactate dehydrogenase. BM, bone marrow; RBC,red blood cell.
